# Workmen's Payments to Sunderland Hospitals

**Published:** 1888-09-01

**Authors:** A. W. Webster

**Affiliations:** Chairman of Workmen's Governors of Sunderland Infirmary.


### Workmen's Payments to Sunderland Hospitals.

By A. W. Webster, Chairman of Workmen's Governors of Sunderland Infirmary.

As our system of collecting weekly subscriptions from workmen has received so much prominence in the recent discussion on "The Pay System in Hospitals," the readers of The Hospital will, perhaps, value the following more extended statement of the results of the methods employed in Sunderland than has yet appeared in its columns. The figures quoted are for workmen's contributions to *medical charities alone* from July 1st, 1887, to June 30th, 1888, and will supply material for comparing the results of a year's work where other methods are in force. The town population may be taken at 130,000.

| | £ | s. | d. |
|---|---|---|---|
| Workmen's subscriptions to Sunderland Infirmary* ... ... ... ... ... ... | £2,842 | 13 | 2 |
| Workmen's Friendly and other society subscriptions to ditto ... ... ... ... | 66 | 5 | 0 |
| Workmen's subscriptions towards a new wing now building at ditto ... ... ... ... | 296 | 3 | 0 |
| Goods sold from workmen's stall at bazaar for ditto | 92 | 0 | 0 |
| Members' subscriptions to Provident Dispensary (this is really the out-patient department of the infirmary, the work of which is done in most hospitals for nothing) ... ... ... ... | 592 | 4 | 6 |
| Workmen's subscriptions to Monkwearmouth Dispensary and Accident Home* ... ... | 340 | 18 | 8 |
| Workmen's Friendly and other society subscriptions to Monkwearmouth Dispensary and Accident Home* ... ... ... ... ... ... | £5 | 5 | 0 |
| Workmen's subscriptions to Sunderland Eye Infirmary* ... ... ... ... ... ... | 17 | 6 | 9 |
| Workmen's Friendly and other society subscriptions to ditto ... ... ... ... | 5 | 5 | 0 |
| | £4,258 | 11 | 9 |

In addition to above £150 2*s.* 8*d.* was raised by the workmen's agency on Hospital Saturday, a good proportion of which would come from workpeople themselves. Payments by patients amount to about £120 in the institutions marked thus *, but are not included in my figures.

The totals may be summarised as follows:—

| | £ | s. | d. |
|---|---|---|---|
| Raised by the penny-a-week system ... ... | £3,497 | 1 | 7 |
| Raised through workmen's societies ... ... | 76 | 15 | 0 |
| Raised by workmen's gifts of goods to bazaar ... | 92 | 0 | 0 |
| Raised by workmen's agency on Hospital Saturday ... ... ... ... ... | 150 | 2 | 8 |
| Members' subscriptions to Provident Dispensary (from 1*d.* upwards) ... ... ... ... | 592 | 4 | 6 |
| | £4,408 | 3 | 9 |

